# Microbial alpha diversity in the intestine negatively correlated with disease duration in patients with Meniere’s disease

**DOI:** 10.1038/s41598-024-83367-7

**Published:** 2024-12-30

**Authors:** Fumihiro Mochizuki, Manabu Komori, Jun Shimizu, Yoshiyuki Sasano, Yusuke Ito, Michael E. Hoffer, Yoshishige Miyabe, Izumi Koizuka

**Affiliations:** 1 Department of Otolaryngology, St. Marianna University School of Medicine, 1-16-2 Sugao, Miyamae-ku, Kawasaki City, 216-8511 Kanagawa Japan; 2 Department of Otolaryngology, University of Miami Miller School of Medicine, Miami, USA; 3 Department of Immunology and Parasitology, St. Marianna University School of Medicine, Kawasaki, Japan; 4 Department of Otolaryngology, Jikei University School of Medicine, Tokyo, Japan

**Keywords:** Microbiology, Diseases, Medical research, Neurology

## Abstract

Ménière’s disease (MD) is characterized by loss of balance and hearing disorders. Although there is known to be endolymphatic hydrops involved in the pathological process, the pathogenesis of the disease is still largely unclear. Approximately half of patients with MD suffer from depressive symptoms and high levels of several stress hormones were observed in MD and depression, simultaneously. Recently, gut microbes have been shown to influence the function of the central nervous system in humans through their metabolites. We studied the intestinal microbiota of 10 patients with MD and 11 healthy donors (HD). Significant negative correlations were found between disease duration and alpha diversity indexes of gut microbes in patients with MD. The relative abundance of the species *Butyricicoccus* ambiguous taxa was increased in patients with MD compared with that of HD. In contrast, *Oscillospiraceae UCG-002/UCG-005* ambiguous taxa and *Anaerovoracaceae* (*Eubacterium*) brachy group uncultured bacterium were increased in the relative abundance of HD than that of patients with MD. The relative abundance of the *Butyricicoccus* species was positively correlated with disease duration. Thus, these compositional alterations of gut microbes in patients with MD are associated with inner ear pathologies, such as endolymphatic hydrops, by changing the metabolite profiles in the intestine.

**Supplementary Information:**

The online version contains supplementary material available at 10.1038/s41598-024-83367-7.

## Introduction

Ménière’s disease (MD) is an intractable disorder characterized by paroxysms of intense rotatory vertigo and auditory symptoms such as aural fullness and hearing loss^1^. The annual prevalence of MD is approximately 38 per 100,000 population and female dominance was observed in Japan^2^. MD is frequently comorbid with obesity, arthritis, chronic fatigue syndrome, hay fever, rhinitis, eczema, drug allergy, irritable bowel syndrome, gastro-esophageal reflux disease, migraine, and psoriasis^3^.

Contrast-enhanced magnetic resonance imaging (MRI) demonstrated that endolymphatic hydrops (EH) were observed in 190 of 205 (95%) ears with symptoms related to MD^4^. In addition, 29 of 45 (65%) asymptomatic contralateral ears exhibited EH on the enhanced MRI, suggesting that EH of the vestibule and cochlea plays a role in the pathogenesis of MD^4^.

In the psychiatry comorbidities of MD, depressive status is prevalent^5,6^. A prospective study^5^ and a systematic review^6^ revealed that 60.4% and 45.9% of patients with MD were depressive, respectively, with poor responses to the surgical treatment^6^. In an exploratory study of MD patients with EH, depression scale scores were correlated with the grade of hearing loss^7^. Furthermore, the ratios of endolymphatic space/total fluid space of the inner ear evaluated by enhanced MRI were correlated with stress response scale scores in patients with MD^7^.

MD is then thought to be associated with an alteration of systemic and tissue fluid homeostasis. The concentration of vasopressin in the plasma and expression of vasopressin receptor 2/aquaporin 2 in inner ear tissues were significantly higher in patients with MD than those in controls^8,9^. The endolymphatic sac drainage surgery significantly decreased the levels of vasopressin in the plasma of patients with refractory MD compared with those in patients with chronic otitis media who underwent tympano-mastoidectomy^10^. Thus, these data suggest a possible role of psychological and physical stressors in the development of MD.

Exposure to the stresses frequently activates the hypothalamus-pituitary-adrenal axis to provide prompt and accurate responses to environmental stimulation^11^. For instance, levels of both vasopressin and cortisol in the plasma were elevated in patients with depression^12^. In addition, the concentration of cortisol in both serum and saliva was significantly increased in patients with MD than those in healthy donors (HD)^13^.

Recent studies have demonstrated that intestinal microbiota interacts with the central nervous system (CNS), referred to as the gut-brain axis, through the hypothalamus-pituitary-adrenal axis function and gut microbe metabolites, such as serotonin and short-chain fatty acids (SCFAs)^14^, some of which were shown to have effects on disease phenotypes of stressed animals^15,16^. In the gut microbiota-related elements, SCFAs, such as acetate, propionate, and butyrate, are considered to be closely involved in the host’s psychological condition^14^. A large cohort data of 1,070 individuals also demonstrated that the abundance of several SCFA-producing bacteria was associated with depression metadata and quality of life scores^17^. Moreover, Oral SCFA supplementation, in a clinical trial, ameliorated the cortisol responses to psychosocial stress of healthy individuals^18^.

However, the relationship between MD and intestinal microbiota still remains unknown. Thus, studying the intestinal microbiota in patients with MD could lead to understanding the pathogenesis of MD and develop a new therapeutic approach. In this study, we studied if changes in the intestinal microbiota might affect the inner ear function of patients with MD.

## Results

### Clinical parameters in patients with MD

The age, sex, and body mass index (BMI) were no significant differences between MD and HD (Table 1). Also, the clinical and metagenomic parameters of patients with MD were shown (Table 1). The mean disease duration for patients with MD in this study was 5.6 ± 3.8 years.

**Table 1:** Demographic and clinical, data of patients with Ménière’s disease (MD) and healthy donors (HD).

	MD (*n* = 10)	HD (*n* = 11)	*P* value
Age	47.8 ± 7.5	43.6 ± 9.2	0.36
Male:female	7:3	7:4	1.00
Body Mass Index	24.3 ± 4.5	25.5 ± 6.9	0.85
Disease duration (year)	5.6 ± 3.8	Not applicable	
Mean hearing threshold (dB)	37.6 ± 15.0	Not applicable	
Dizziness Handicap Inventory score	40.6 ± 19.2	Not applicable	

The dizziness handicap inventory (DHI) is a standard questionnaire that quantitatively evaluates the degree of handicap in the daily life of patients with vestibular disorders; it consists of 25 questions^19^, such as “Because of your problem, do you feel frustrated?”, “Does your problem interfere with your job or household responsibilities?”, and “Does turning over in bed increase your problem?”^19^. A previous study reported that the mean DHI scores of 29 definite MD cases were 53.79 and 40.34 before and after treatment, respectively^20^. The mean DHI score for patients with MD in this study was 40.6 ± 19.2 (Table 1).

The hearing assessment used a quadrant method is the mean of the hearing threshold at the frequencies of 500, 1000, 2000, and 4000 Hz by the pure tone audiogram. In a study of 143 patients with definite MD who had their hearing measured on the affected side, the mean hearing threshold using a quadrant method was 41.84 ± 15.7 dB^21^. The affected-sided mean hearing threshold used a quadrant method for patients with MD in this study was 37.6 ± 15.0 dB (Table 1). Thus, these data suggested that this cohort study enrolled patients with mild to moderate MD.

### Alpha diversity index scores and beta diversity indices

Alpha diversity is defined as the diversity within a microbial community^22^. Inverse associations between body weights and alpha diversity index scores were observed only in female HD^23^ and female patients with schizophrenia^24^. Alpha diversity index scores tended to decrease in patients with Crohn’s disease compared with HD^25,26^. Previous studies demonstrated inconsistent results of the scores in patients with depression^27^. Our study couldn’t find any significant differences between MD and HD in alpha index scores (Table 2). We suggest that the comparable numbers of operational taxonomic units and alpha diversity index scores in the gut microbes of this study between MD and HD may ensure the validity of statistical comparison of the relative abundance of bacterial taxa in the following result section to some extent.

**Table 2:** Alpha diversity index scores of patients with Ménière’s disease (MD) and healthy donors (HD).

	MD (*n* = 10)	HD (*n* = 11)	*P* value
Observed operational taxonomic unit number	185.2 ± 53.2	210.6 ± 65.2	0.38
Shannon index score	5.93 ± 0.52	6.17 ± 0.41	0.38
Faith phylogenetic diversity score	15.2 ± 3.2	18.2 ± 3.7	0.22

**Fig. 1:**
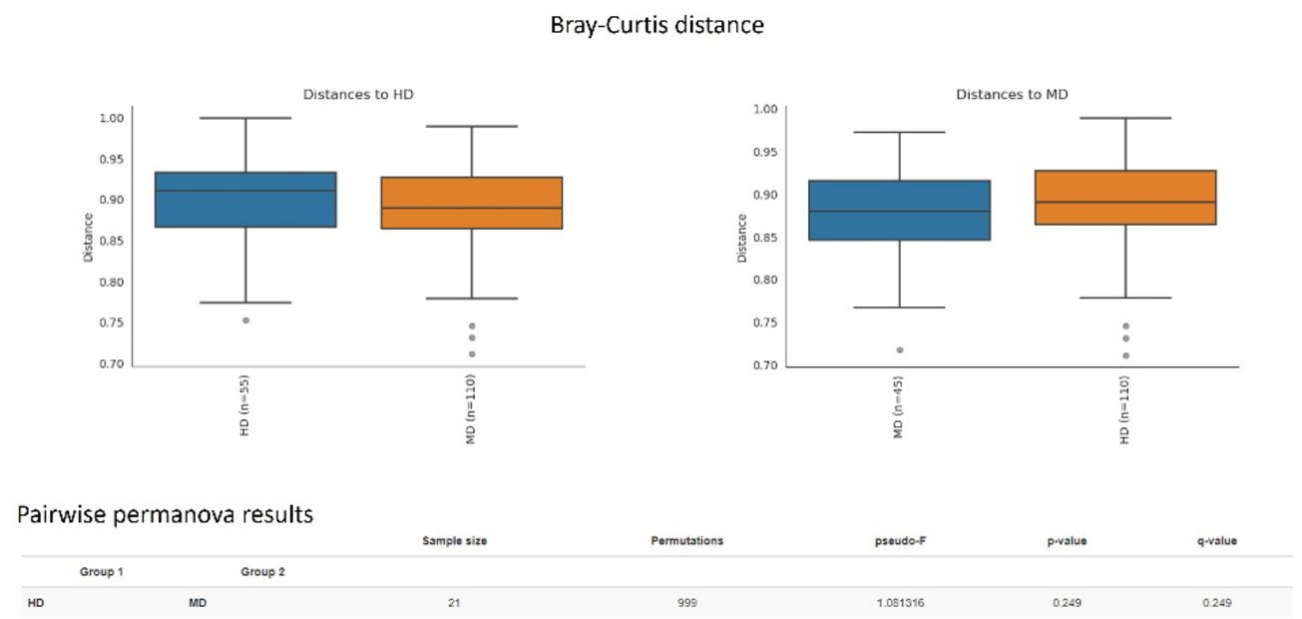
Measuring beta diversity distance between MD and HD. We did not obtain a significant finding between MD and HD in Bray-Curtis distance.

Beta diversity is defined as the distances among microbial communities^22^. We did not find any significant distances between MD and HD in the beta diversity analyses (Fig. 1 and Supplement Fig. 1).

### Comparison among alpha diversity indexes and clinical parameters

**Fig. 2:**
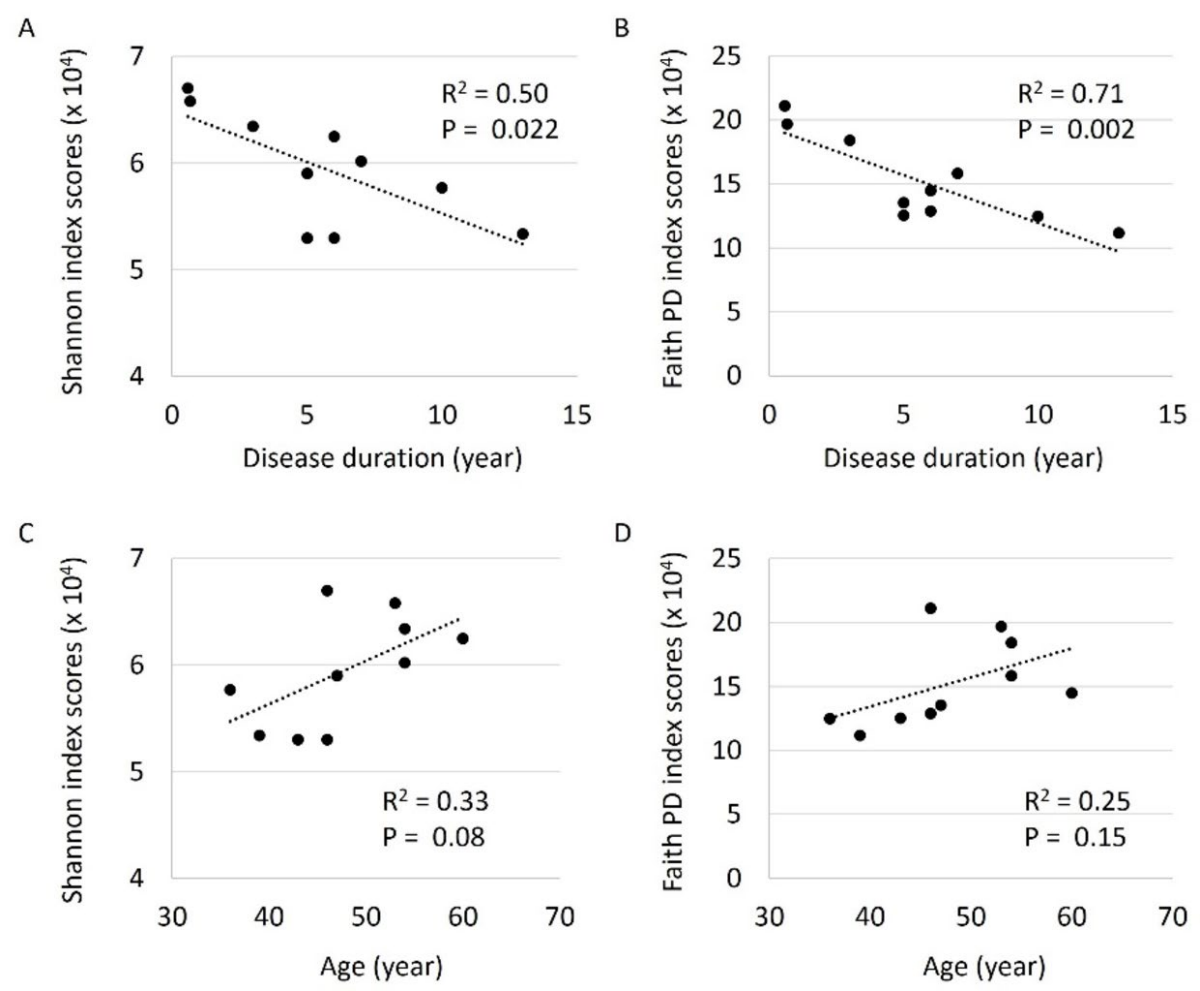
Comparison between alpha diversity index scores and disease duration/age of patients with MD. (**A**) The Shannon index: A significant negative correlation was observed (R^2^ = 0.50, *P* = 0.022). (**B**) The Faith phylogenetic diversity index: A significant negative correlation was observed (R^2^ = 0.71, *P* = 0.002). (**C**, **D**) We did not find relationships between patient age and alpha diversity index scores.

We next compared the alpha diversity index scores of this study with clinical parameters in patients with MD. Negative correlations were observed between disease duration and Shannon/Faith phylogenetic diversity (PD) index scores (R^2^ = 0.50, *P* = 0.022 and 0.71, *P* = 0.002, respectively) (Fig. 2A, B). Patient age (Fig. 2C, D), DHI scores and mean hearing threshold did not significantly correlate with alpha diversity index scores.

### Comparison of relative abundance of bacterial taxa between MD and HD

**Fig. 3:**
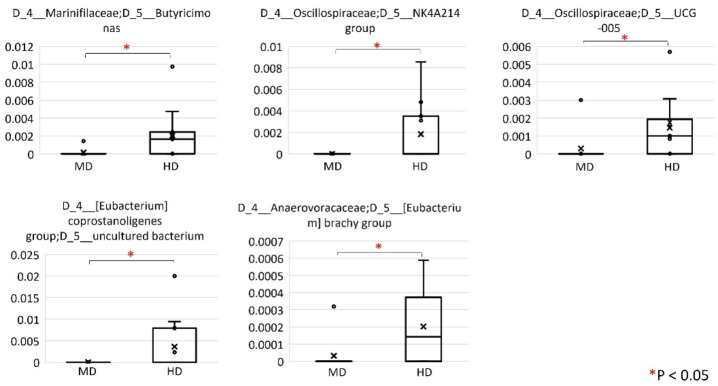
Comparison of relative abundance of bacterial taxa between MD and HD at level 6 (the genus level). In this level, the genera *Butyricimonas*, *Oscillospiraceae NK4A214* group, *Oscillospiraceae UCG-005*, and *Anaerovoracaceae* (*Eubacterium*) brachy group were increased in the relative abundance of HD compared to those of patients with MD.

**Fig. 4:**
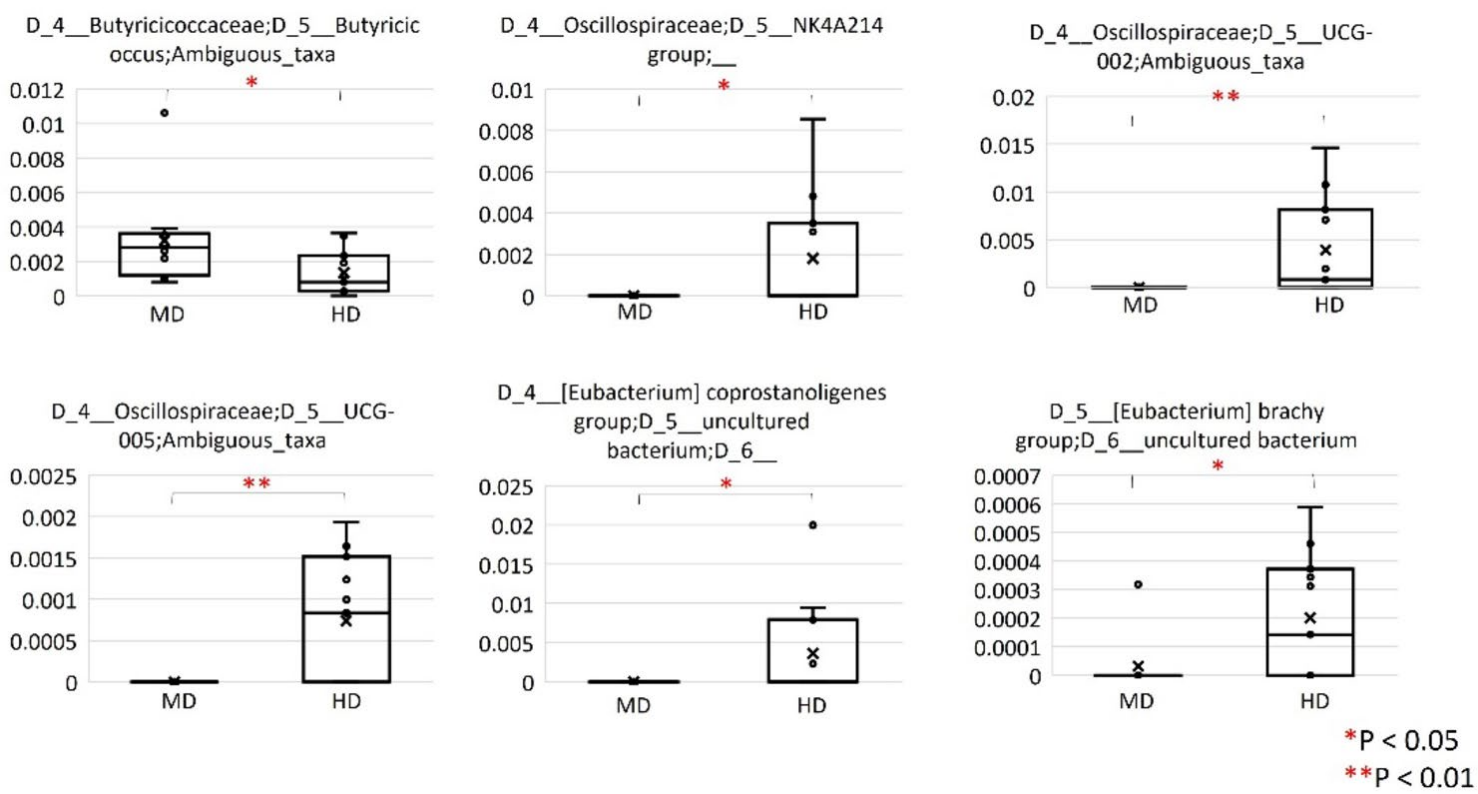
Comparison of relative abundance of bacterial taxa between MD and HD at level 7 (the species level). In this level, the species *Butyricicoccus* ambiguous taxa was increased in the relative abundance of patients with MD compared with that of HD. *Oscillospiraceae UCG-002* ambiguous taxa, *Oscillospiraceae UCG-005* ambiguous taxa, and *Anaerovoracaceae* (*Eubacterium*) brachy group uncultured bacterium were increased in the relative abundance of HD compared to those of patients with MD.

**Fig. 5:**
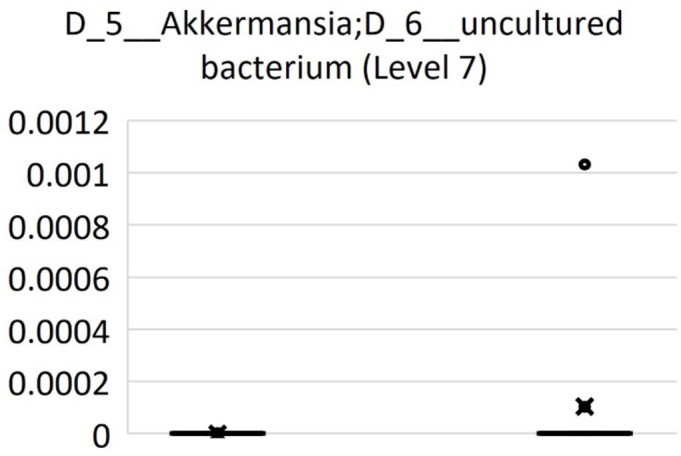
Comparison of relative abundance of *Akkermancia* at level 7 between patients with MD and HD. The genus *Akkermancia* was not observed in the gut microbiota of patients with MD.

We compared the relative abundance of bacterial taxa between MD and HD using QIIME2’s relative feature tables at levels 6 (the genus level) and 7 (the species level). At level 6, the genera *Butyricimonas*, *Oscillospiraceae NK4A214* group, *Oscillospiraceae UCG-005*, (*Eubacterium*) *coprostanoligenes* group uncultured bacterium, and *Anaerovoracaceae* (*Eubacterium*) brachy group were increased in the relative abundance of HD compared to those of patients with MD (Fig. 3). At level 7, the species *Butyricicoccus* ambiguous taxa was increased in the relative abundance of patients with MD compared with that of HD (Fig. 4). *Oscillospiraceae UCG-002* ambiguous taxa, *Oscillospiraceae UCG-005* ambiguous taxa, and *Anaerovoracaceae* (*Eubacterium*) brachy group uncultured bacterium were increased in the relative abundance of HD compared with those with patients with MD (Fig. 4). The genus Akkermancia was not observed in the gut microbiota of patients with MD whereas the genus was recognized in two HD (Fig. 5).

### Comparison of predicted metagenomic gene functions between MD and HD

**Fig. 6:**
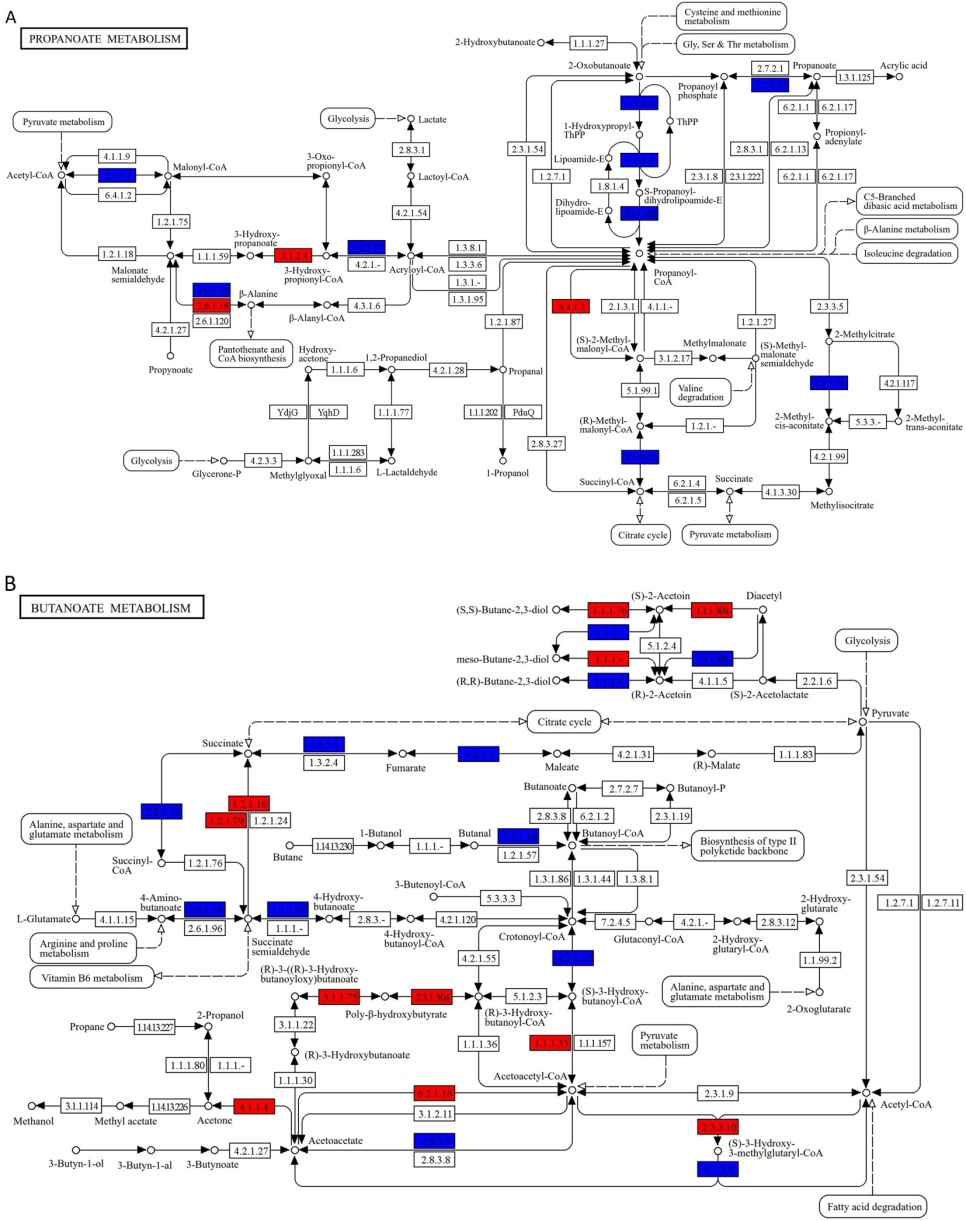
Comparison of predicted gene functions of gut microbes between MD and HD. We identified MD-associated predicted gene functions more prevalent than twice (highlighted with red) or less than half (highlighted with blue) compared with HD. The pathway mapping of the Kyoto Encyclopedia of Genes and Genomes (KEGG) database demonstrated several MD-associated gene functions in propionate (**A**) and butyrate (**B**) metabolism pathways.

We estimated metagenomic gene functions of gut microbes from the taxonomic data using PICRUSt2 in MD and HD^28^. We did not find any significant differences in the gene function after false discovery rate correction (q < 0.05). Alternatively, in an exploratory analysis, we identified MD-associated predicted gene functions as more prevalent than twice or less than half compared with HD. The pathway mapping of the Kyoto Encyclopedia of Genes and Genomes (KEGG) database demonstrated several MD-associated gene functions in propionate (Fig. 6A) and butyrate (Fig. 6B) metabolism pathways.

### Comparison of relative abundance of bacterial taxa with demographic, clinical, and metagenomic parameters

**Fig. 7:**
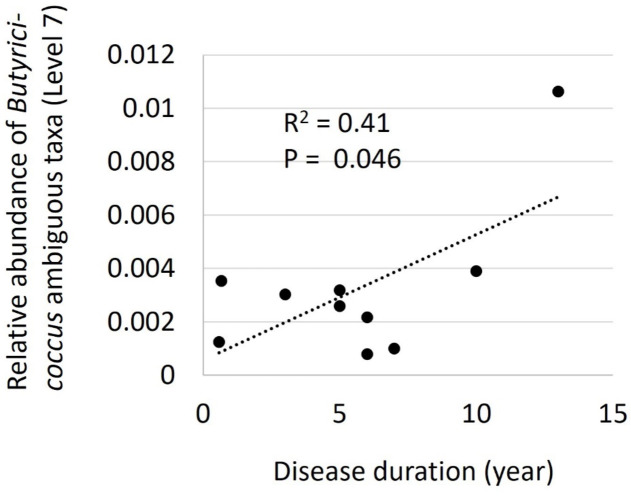
Comparison of relative abundance of *Butyricoccus* species at level 7 and disease duration of patients with MD. The relative abundance was positively correlated with disease duration in patients with MD (R^2^ = 0.41, *P* = 0.046).

We finally compared the relative abundance of bacteria taxa with demographic, clinical, and metagenomic parameters in patients with MD. The relative abundance of *Butyricoccus* at level 7 was positively correlated with the duration of MD disease (R^2^ = 0.41, *P* = 0.046) (Fig. 7).

## Discussion

In our present study, we analyzed gut microbe composition in patients with MD and HD to elucidate the characteristics and functions. We demonstrated significant negative correlations between patients’ disease duration and gut microbe alpha diversity index scores. The relative abundance of the genus *Butyricicoccus* was increased in patients with MD compared with HD. In addition, several genera of the family *Oscillospiraceae*, *Butyricimonas*, and *Anaerovoracaceae* (*Eubacterium*) bacteria were increased in the relative abundance of HD, compared to those in patients with MD. Specifically, a positive correlation between the *Butyricicoccus* species abundance and MD patients’ disease duration was observed.

In the alpha diversity analysis of this study, there were no significant differences between patients with MD and HD. Nevertheless, the Shannon and Faith PD index scores negatively correlated with the disease duration of patients with MD. Previous studies showed that intestinal microbe composition in HD was relatively stable for years^29,30^. Low alpha diversity index scores were observed in patients with colitis compared to HD^25,26,31^. Thus, composition alteration of the intestinal microbiota has progressed with stress accumulation and long disease duration in patients with MD.

The genus *Butyricicoccus* produces butyrate^32^, an SCFA, and has recently received attention for the abundance decrease in patients with inflammatory bowel disease^33,34^. SCFAs, such as acetate, propionate, and butyrate, activate the enteroendocrine cells to produce serotonin^35^ and a gut hormone peptide YY^36^. The activated cells directly stimulated vagal sensory neurons in in vitro experiments through these molecules^37^. The biological mechanisms are suggested to lead to interaction between gut microbiota and CNS function as a gut-brain axis^14,16^. In a mouse model of depression with mild unpredictable stresses, the genus *Butyricicoccus* was one of the significantly decreased intestinal bacteria and the abundance was correlated with serum serotonin concentration, both of which were increased in the administration of an antidepressant^38^. In our study, the relative abundance of the genus *Butyricicoccus* was positively correlated with disease duration in patients with MD, suggesting the protective role of the genus in slowing disease progression.

The family *Oscillospiraceae* contains butyrate-producing species^39^ and *Oscillospiraceae UCG 003* and *UCG 002* were reported as depression-associated bacteria in a multicohort analysis^40^. In the research, 8 of 24 studies demonstrated significantly decreased alpha diversity index scores in patients with depression compared with non-depressive groups.

In a systematic review, low and high abundance of the families *Oscillospiraceae* and *Eubacteriaceae*, respectively, were observed in individuals with depression^41^. Another systematic review reported that the relative abundance of the genus *Butyricimonas* was related to major depressive disorder and generalized anxiety disorder^27^. A low abundance of the genus *Butyricimonas* was found in patients with both irritable bowel syndrome, a common comorbidity of MD^3^, and depression^42^. Several species from the genera *Eubacterium*^43^ and *Butyricimonas*^44^ are associated with SCFA production in the human intestine.

Based on these data, the altered composition of gut microbiota of this study, such as *Butyricicoccus* and *Oscillospiraceae*, may play an essential role in the pathogenesis of MD probably through their SCFA production.

Obesity, another common morbidity of MD^3^, elevated the risk of developing depression in women and eating disorders are associated with both obesity and depression^45^. High-fat diet-induced obesity and anxiety-like behavior with gut microbe compositional alteration in female mice^46^. Several human studies of gut microbiota demonstrated that a relative abundance of MD-related taxa, *Butyricimonas* and *Oscillospiraceae NK4A214*, were altered in the intestine of obesity^47,48^. Interestingly, a disease-associated genus in obesity, the genus *Akkermancia*^48,49^ was not present in the intestine of patients with MD, although there was no significant difference in the relative abundance between MD and HD. The genus *Akkermancia* has been reported to increase in the gut microbiota at the animal level with walking exercise^50^. Daily lifestyle guidance for MD generally includes improving sleep, diet, exercise, and avoidance of stress^51,52^ These data may suggest that several gut microbes in the intestine of patients with MD provide a molecular basis of not only MD but also the major complications, such as depression and obesity.

There are several limitations in this assessment as a retrospective cross-sectional study. The number of recruited patients with MD was small, so this may indicate that the patients were representative of that region. The longitudinal effects of the therapies in the patients with MD are still unknown. Further longitudinal clinical studies are needed to elucidate the relationships between gut microbiota and important clinical features of MD, such as disease onset and acute aggravation.

## Conclusions

We hypothesize that gut microbiota may play a role in MD pathogenesis through these metabolic interactions. Thus, targeting the gut microbiota could provide insights into MD and support the development of new therapeutic strategies. Further research on MD and gut microbiota is warranted.

## Methods

### Participants

**Fig. 8:**
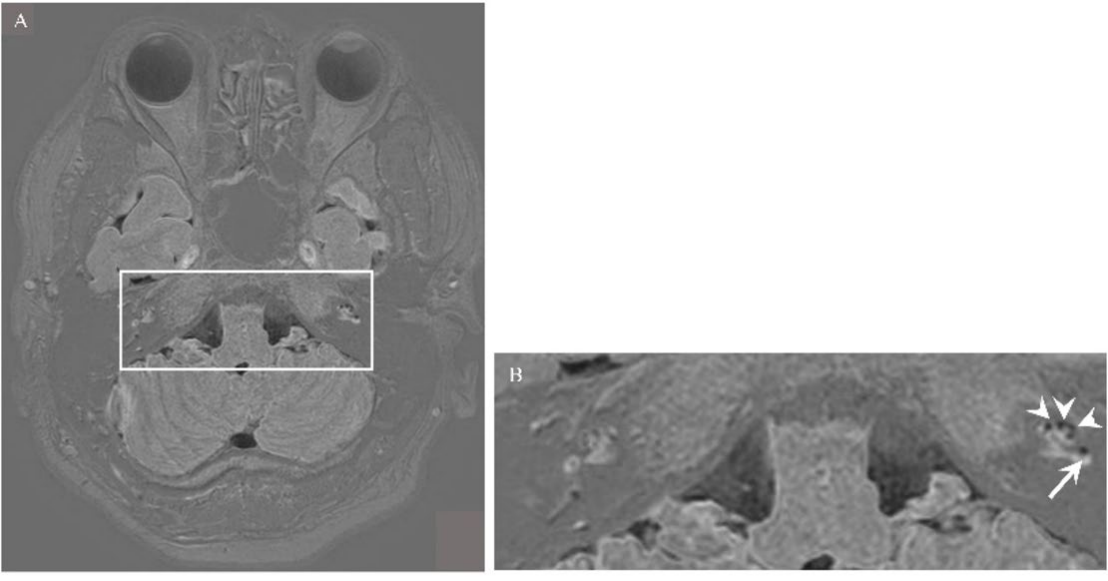
(**A**) An axial view of the image by contrast-enhanced magnetic resonance of the inner ear in patients with MD. (**B**) A magnified view of the inset of (**A**) and indicates endolymphatic hydrops (EH) of the diseased left inner ear in patients with MD. The arrowheads indicate EH of the cochlea, and the arrows indicate the EH of the vestibule. Significant EH is shown in the ear with symptoms, but not in the contralateral ear.

The participants are 11 HD (7 males and 4 females, mean age 43.6 ± 9.2 years) and 10 patients (7 males, 3 females; mean age 47.8 ± 8.1 years) with unilateral definite MD diagnosed according to the diagnostic criteria of the Barany Society, and all patients had significant endolymphatic hydrops on the affected side on enhanced MRI of the inner ear. We show representative enhanced MRI images in this study (Fig. 8). The participants did not have any psychiatric, diabetic, or gastrointestinal diseases.

This study was approved by the Clinical Trials Subcommittee (Observational Studies) of St. Marianna University School of Medicine. The approval number is 5674. Informed consent was given to the subjects in writing and orally. According to the principles of the Declaration of Helsinki, the subject’s informed consent was obtained from all subjects and/or their legal guardian(s). The information obtained was anonymized so that individual patients could not be identified. All methods were carried out following relevant guidelines and regulations.

### Clinical tests

The clinical data performed closest to the intestinal microbiota sample collection were used for this study. DHI score is a standard questionnaire that quantitatively evaluates the degree of handicap in the daily life of patients with vestibular disorders; it consists of 25 questions^19^. The total score ranges from 0 (no disability) to 100 (severe disability). DHI score is classified as no impairment (0–14 points), mild (16–25 points), moderate (28–44 points), and severe (46 points or more)^19^. The audiometry is that pure tone audiogram thresholds were measured at octave intervals between 125 Hz and 8 kHz. The mean hearing threshold that was compared used a quadrant method in which the hearing thresholds at frequencies of 500, 1,000, 2,000 Hz, and 4000 Hz were a, b, c, and d dB, respectively, and the threshold (dB) was calculated using the formula (a + b + c + d)/4.

### Fecal sampling, DNA extraction, and sequencing

The participants collected fecal samples during a period without any abdominal discomfort. Fecal samples were collected using Cykinso fecal collection kits containing guanidine thiocyanate solution (Cykinso Inc., Tokyo. Japan) and stored at 4 °C. DNA extraction from the fecal samples was performed using an automated DNA extraction machine (GENE PREP STAR PI-480; Kurabo Industries Ltd., Osaka). The V1-V2 region of the 16 S rRNA gene was amplified using a forward primer (16S_27Fmod: TCG TCG GCA GCG TCA GAT GTG TAT AAG AGA CAG AGR GTT TGA TYM TGG CTC AG) and reverse primer (16S_338R: GTC TCG TGG GCT CGG AGA TGT GTA TAA GAG ACA GTG CTG CCT CCC GTA GGA GT) with the KAPA HiFi Hot Start Ready Mix (Roche). To sequence the 16 S amplicons using the Illumina MiSeq platform, dual index adapters were attached using the Nextera XT Index kit. The DNA concentration of the mixed libraries was quantified by qPCR using KAPA SYBR FAST qPCR. The master mix (KK4601, KAPA Biosystems) was used with primer 1 (AAT GAT ACG GCG ACC ACC) and primer 2 (CAA GCA GAA GAC GGC ATA CGA). Library preparations were performed according to the Illumina 16 S library preparation protocol (Illumina, San Diego, CA, USA). Libraries were sequenced using the MiSeq Reagent Kit v2 (500 cycles) and 250-bp paired ends.

### Taxonomy assignment based on 16 S rRNA gene sequences

The paired-end reads of the partial 16 S rRNA gene sequences were analyzed using QIIME2 (version 2020.8). The steps for data processing and assignment based on the QIIME2 pipeline were as follows: (1) DADA2 was used for joining paired-end reads, filtering, and denoising; (2) taxonomic information was assigned to each ASV by using a naive Bayes classifier in the QIIME 2 classifier with the 16 S gene of the V1-V2 region data of SILVA (version 138) to determine the identity and composition of the bacterial genera. The reliability of these tests has been reported previously^53,54^.

We obtained alpha diversity index scores, such as observed operational taxonomic unit numbers, Shannon index scores, and Faith PD scores, and beta diversity indices, such as Bray-Curtis, Jaccard, and UniFrac distances, in HD and MD.

Using the taxonomic data, we performed a functional annotated analysis of gut microbes by PICRUSt2 software^28^. We obtained alpha and beta diversity index scores of the individuals using the same software. We evaluated significant differences in the metagenomic gene function of gut microbes by PICRUSt2 software. As an alternative approach for the comparison of the gene function between MD and HD, an Excel software table obtained from PICRUSt2’s feature table was calculated with false discovery rate. We identified MD-associated predicted gene functions as more prevalent than twice or less than half compared with HD and visualized the results by the pathway mapping of the Kyoto Encyclopedia of Genes and Genomes (KEGG) database^55–57^.

### Statistical analysis

We compared clinical parameters, such as age, disease duration, DHI scores, and BMI, and laboratory parameters, such as audiometry results and alpha diversity index scores between HD and MD using Wilcoxon rank test (two-tailed). We compared gender of the two groups with Figher’s exact test (two-tailed). Statistical analysis of correlations among the parameters used linear regression analysis (two-tailed). We used software SPSS 28.0 (IBM Corp. Armonk, NY, USA) for the statistical analysis of this study. Each value was expressed as mean ± standard deviation (s.d.).

## Electronic Supplementary Material

Below is the link to the electronic supplementary material.

Supplementary Material 1

## Data Availability

Data availability statement: The datasets generated during the current study are available in the DDBJ repository, accession number BioProject: PRJDB17474 or https://ddbj.nig.ac.jp/resource/bioproject/PRJDB17474.
