# Supplementary material for: Microbial alpha diversity in the intestine negatively correlated with disease duration in patients with Meniere’s disease
Source: Sci Rep. 2024 Dec 30;14:31893. doi: 10.1038/s41598-024-83367-7 (PMC11686369; doi:10.1038/s41598-024-83367-7)
Supplement: Supplementary file 1 — Supplementary Material 1 [file 41598_2024_83367_MOESM1_ESM.pdf]

Supplement Figure 1.

Measuring beta diversity distances between MD and HD. We did not obtain a significant finding between MD and HD in Jaccard, unweighted and weighted UniFrac distances.

A

Jaccard distance

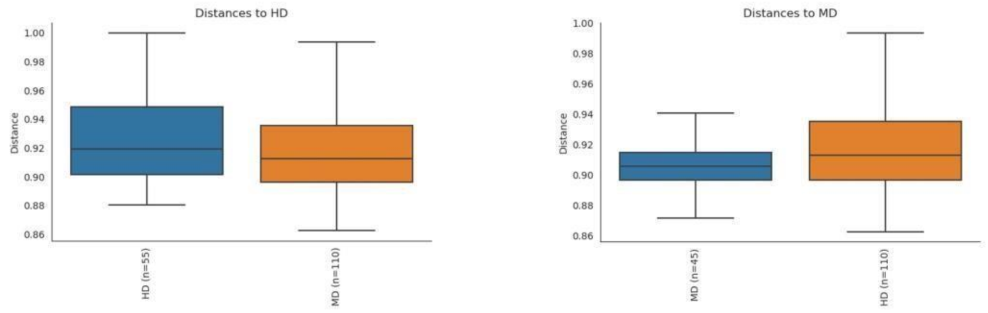

Pairwise permanova results

Group 1	Group 2	Sample size	Permutations	pseudo-F	p-value	q-value
HD	MD	21	999	1.017973	0.318	0.318

Supplement Figure 1A

B

Unweighted UniFrac distance

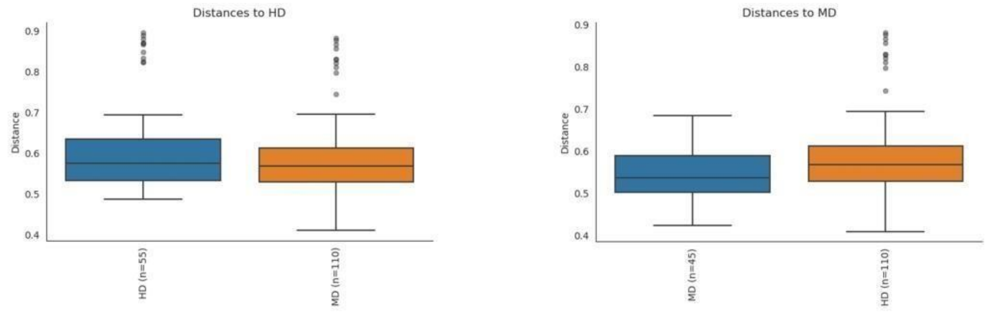

Pairwise permanova results

Group 1	Group 2	Sample size	Permutations	pseudo-F	p-value	q-value
HD	MD	21	999	0.969725	0.483	0.483

Supplement Figure 1B

C

Weighted UniFrac distance

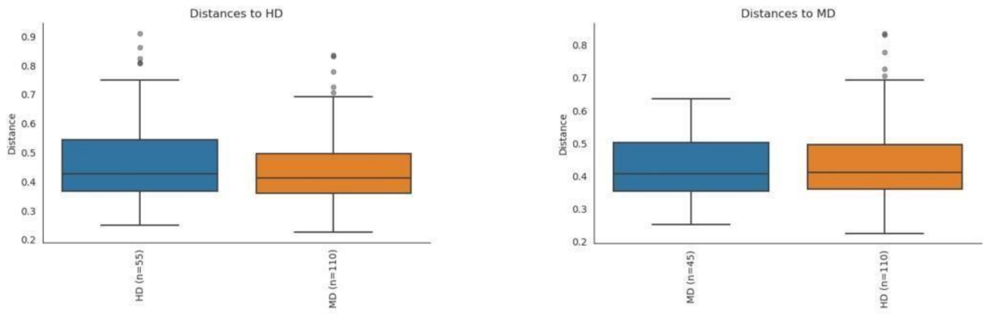

Pairwise permanova results

		Sample size	Permutations	pseudo-F	p-value	q-value
Group 1	Group 2					
HD	MD	21	999	0.323543	0.973	0.973

Supplement Figure 1C
